# Breast Milk and the Importance of Chrononutrition

**DOI:** 10.3389/fnut.2022.867507

**Published:** 2022-05-12

**Authors:** Mario Daniel Caba-Flores, Angel Ramos-Ligonio, Alberto Camacho-Morales, Carmen Martínez-Valenzuela, Rubí Viveros-Contreras, Mario Caba

**Affiliations:** ^1^Centro de Investigaciones Biomédicas, Universidad Veracruzana, Xalapa, Mexico; ^2^LADISER Inmunología y Biología Molecular, Facultad de Ciencias Químicas, Universidad Veracruzana, Orizaba, Mexico; ^3^Unidad de Neurometabolismo, Centro de Investigación y Desarrollo en Ciencias de la Salud, Universidad Autónoma de Nuevo León, San Nicolás de los Garza, Mexico; ^4^Unidad de Investigación en Ambiente y Salud, Universidad Autónoma de Occidente, Los Mochis, Mexico

**Keywords:** chrononutrition, melatonin, glucocorticoids, circadian feeding, tryptophan, cannabinoids, oligosaccharides, secretory IgA

## Abstract

During pregnancy the human fetus receives timed cues from the circadian rhythms of temperature, metabolites, and hormones from the mother. This influence is interrupted after parturition, the infant does not secrete melatonin and their circadian rhythms are still immature. However, evolution provided the solution to this problem. The newborn can continue receiving the mother's timed cues through breastmilk. Colostrum, transitional, and mature human milk are extraordinary complex biofluids that besides nutrients, contain an array of other non-nutritive components. Upon birth the first milk, colostrum, is rich in bioactive, immunological factors, and in complex oligosaccharides which help the proper establishment of the microbiome in the gut, which is crucial for the infants' health. Hormones, such as glucocorticoids and melatonin, transfer from the mother's plasma to milk, and then the infant is exposed to circadian cues from their mother. Also, milk components of fat, proteins, amino acids, and endogenous cannabinoids, among others, have a markedly different concentration between day and night. In the present review, we give an overview of nutritive and non-nutritive components and their daily rhythms in human milk and explore their physiological importance for the infant. Finally, we highlight some interventions with a circadian approach that emphasize the importance of circadian rhythms in the newborn for their survival, proper growth, and development. It is estimated that ~600,000 deaths/year are due to suboptimal breastfeeding. It is advisable to increase the rate of exclusive breastfeeding, during the day and night, as was established by the evolution of our species.

## Introduction

Being active and eating during typical times of rest, as with night-shift work, disrupts circadian clocks and is related to a higher risk of several metabolic disorders (1). Recent studies have also found that restricting eating to certain times of day can be beneficial to health (2), and from these observations the field of chrononutrition has developed (3). The goal of chrononutrition is to adjust nutrition quality and intake to coordinate with an individual's biological clock, so that one consumes the optimal type and quantity of food at the correspondingly optimal time of day (3). Most of the current understanding of chrononutrition arose from work in adults and thus does not encompass the lifespan of developing humans. Rest and activity patterns and nutritional needs change as humans develop. Unlike adults, newborns ingest milk during the day and night, and this has an important biological significance: the concentration of milk components changes according to the circadian rhythms of the mother. Moreover, the infant is sensitive to milk and environmental circadian cues. In the present contribution, we review the current evidence about these two topics related to rhythms in breastmilk and environmental conditions and discuss their relevance for the proper establishment of the infant's circadian rhythms. Some studies label the temporal changes of milk components as circadian but, in most cases, they describe only different concentrations between specific hours of the day and night; in the present review, we will refer to them as daily rhythms. Our main emphasis will be on human studies.

In the womb, the fetus is exposed for approximately 9 months to the circadian, physiological, metabolic, and behavioral rhythms of the mother. This circadian milieu is abruptly interrupted upon birth, but nature developed the perfect substitution: maternal milk whose composition changes according to the circadian rhythms of the mother (4–7). Not surprisingly, in humans the newborn ingests milk during the day and the night, and the nutritive and non-nutritive components change accordingly. The world health organization recommends exclusive breastfeeding for at least the first 6 months of age to improve child survival, healthy growth, and development but unfortunately, breastfeeding rates in the world are low (8).

## Nutrients in Human Milk Through Lactation

**Figure 1:**
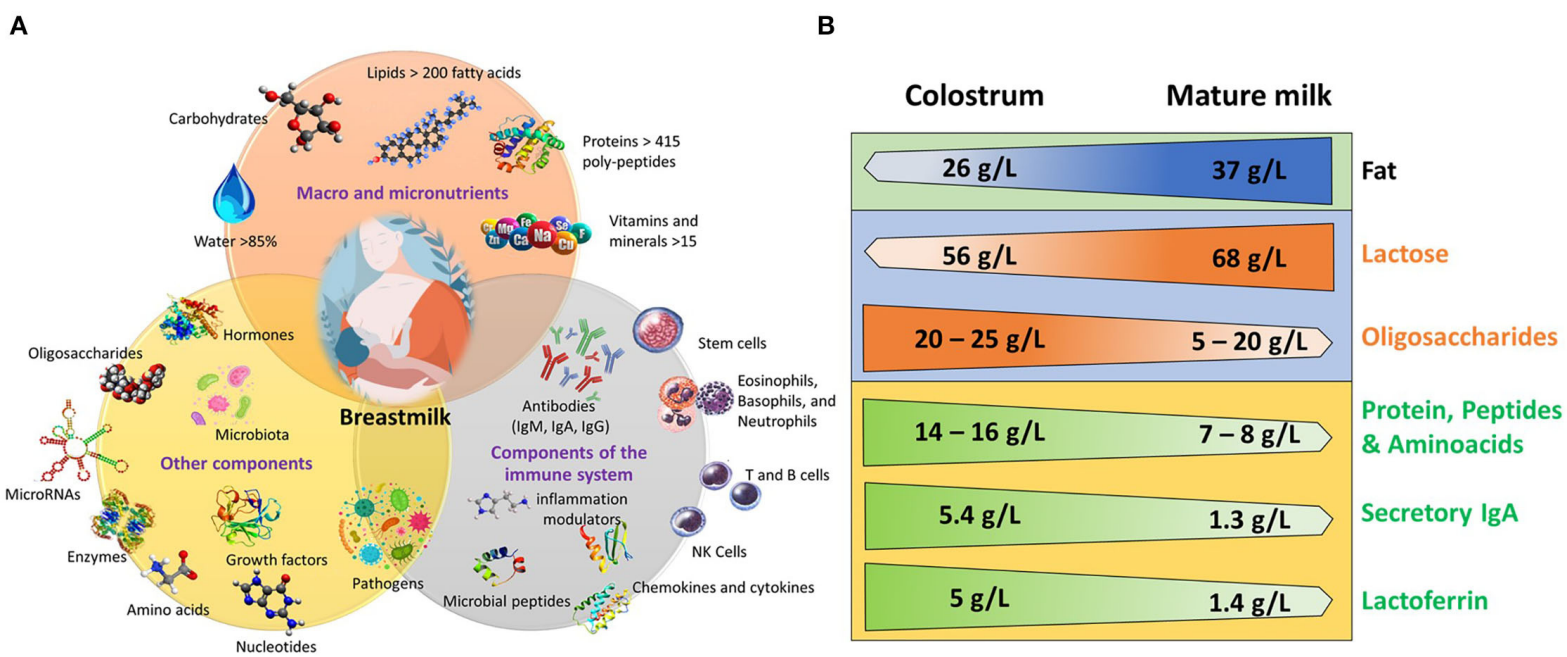
Components of human milk and differences between colostrum and mature milk. **(A)** Macro and micronutrients, components of the immune system, and other components. Modified from (7, 9, 10, 14, 15). **(B)** Concentration of some components in colostrum and in mature milk. Modified from (4, 11–14, 16).

Human milk is an extremely complex biofluid with dynamic composition, beginning with the first milk, colostrum, through transitional and mature milk; it changes significantly depending on the maternal diet, environmental factors, and whether milk is produced for preterm or term infants (9–11). Mature human milk composition contains 88% water, 7% carbohydrates, 4% fat, and 1% protein (9). Protein and total amino acid concentration is highest in colostrum 14–16 g/L, then steadily decreases to 7–8 g/L in mature milk (12). Caseins and whey proteins are the main proteins in milk, but proteomic analysis has identified ~1,700 different proteins (13). Fat concentration has an increasing trend from 26 g/L in colostrum to 37 g/L in mature milk (11). Carbohydrates are the most abundant macronutrient, in which lactose concentration is very stable during lactation with a mean concentration of 61.4 g/L (4), with a lower concentration in colostrum (56 g/L) than in mature milk in which it reaches its highest concentration (68 g/L)to support the increasing growth of the infant [Figure 1; (16)]. Moreover, milk also has vitamins, enzymes and coenzymatic factors, hormones, and immunological factors [(14); Figure 1].

## Non-Nutritive Components and Immunological Factors

The immune system of the newborn is immature and human milk is rich in immunological factors. It contains immunoglobulins, immune cells, complement proteins, and immunomodulatory and antimicrobial factors (17–19). Immunoglobulin A (IgA), Immunoglobulin G (IgG) and Immunoglobulin M (IgM) are present in colostrum, but IgA is different from that found in plasma. It is a dimer with a joining J chain protein and a secretory component named secretory IgA (SIgA), which increases its resistance to proteolytic degradation in the intestine (20). Consistent with this finding, significant quantities of SigA are found in the infant stool, which decreases in parallel with that in milk (21). Levels of sIgA are much larger in colostrum, in comparison with IgG and IgM. Their levels decline from 5.4 g/L in colostrum to 1.3 g/L in mature milk [(17); Figure 1]. Lactoferrin, an iron-binding glycoprotein with bacteriostatic and bactericidal activity against *Escherichia coli*, Candida *albicans*, and other pathogens, such as viruses (22), is higher in colostrum (5 g/L) than in mature milk [1.4g/L; (17); Figure 1]. In addition, human breast milk contains pluripotent stem cells and leucocytes (23–26), plus inflammatory and anti-inflammatory cytokines (27). Polyunsaturated fatty acids, such as docosahexaenoic (DHA) and arachidonic (ARA) acids, have immunomodulatory properties and participate in several developmental and cognitive processes (28). Contrary to the common belief that human milk is sterile, it has been established that in mature milk, the infant consumes ~8 X 10^4^-10^6^ commensal bacteria, fungi, and viruses that colonize the infant gut to form a microbiome that allows proper development of the immune system (9, 29). Also, breastmilk contains ~1,400 species of mature microRNAs that are absorbed by the intestinal epithelial cells and are involved in immunomodulatory and epigenetic regulation (30, 31). Milk also contains a complex array of approximately 200 oligosaccharides, which have important immunological properties and are essential to the development of a “healthy” microbiome. Their concentration is highest in colostrum (20–25 g/L) and decrease through lactation to 5–20 g/L (14, 32). Cannabinoids are among the several neurochemicals found in milk; these include anandamide (AEA) and 2-arachidonoyl glycerol (2-AG), the latter in much larger concentration [(33); Figure 1]. Thus, milk is a complex biofluid that contains myriad nutritive and non-nutritive components. Human milk has an addition allevel of complexity: it reflects circadian rhythms of the mother.

## Diurnal Changes in Milk Components

**Figure 2:**
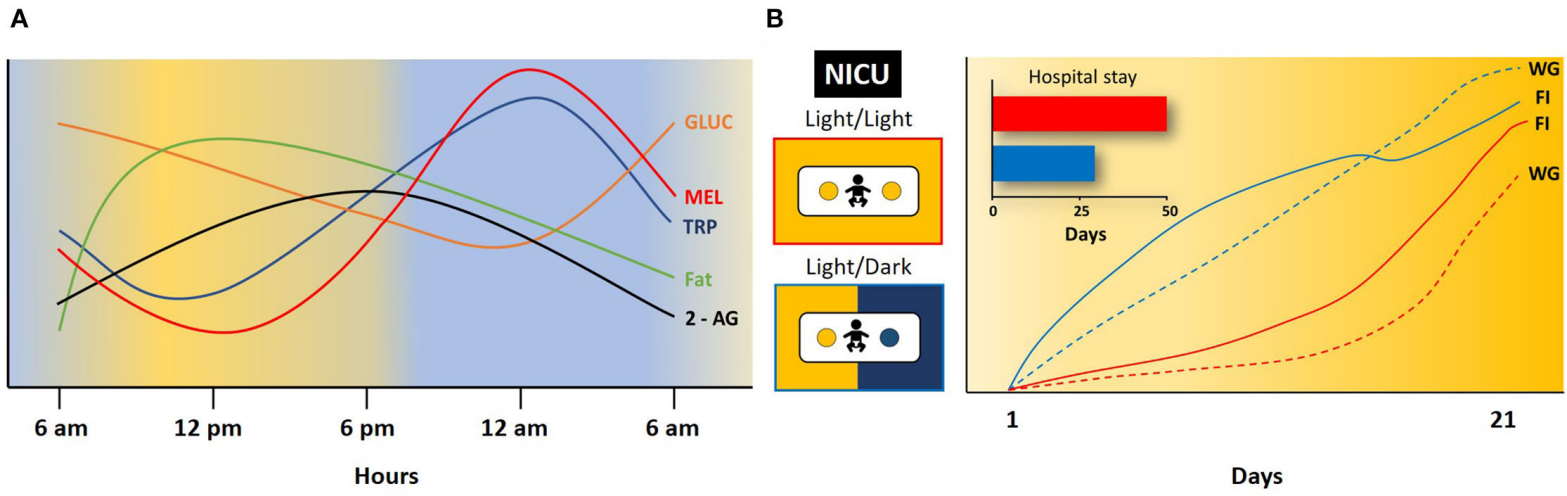
Daily rhythm in some milk components and effect of light/dark conditions on infant development. **(A)** Changes in concentration of glucocorticoids (GLUC), melatonin (MEL), tryptophan (TRP), fat and 2-arachidonoyl glycerol (2-AG) in 24 h. Modified from (5, 6, 33, 35, 36). **(B)** Effect of constant light (Light/Light) or 12 h light and 12 h dark (Light/Dark) on food intake (FI), weight gain (WG), and length of hospital stay in infants at the neonatal intensive care unit (NICU). Blue line and dashed lines infants in Light/Dark; Red line and dashed lines infants in Light/Dark. Note the shorter hospital stay and the increase in food intake and weight gain in infants in Light/Dark cycle in comparison to infants in Light/Light conditions. Modified from (45).

In human milk, there are circadian hormonal variations, including glucocorticoids (GLUC), melatonin, (MEL), Leptin, Ghrelin, and others. Plasma GLUC increases during the last phase of the night, reaches a peak during the morning, and then decreases (34). In contrast, MEL increases during the night and it is almost negligible in plasma during the day (35). GLUC and MEL may communicate “time of day and night” to the body; they are associated with alertness and sleep phases. GLUC and MEL are transferred to breast milk, and their concentration mirrors that in plasma. GLUC concentration in human milk is higher in the morning. MEL levels are low in the evening and the first part of the night, then increase again (5, 36). At 3–4 days after parturition MEL is below the limit of detection between 1,400 and 1,700 h, but during the night between 0200 and 0400 it reaches a concentration of 99 ± 26 pmol/L (6, 35). Concentration in human milk of another hormone, Leptin, is significantly higher during 10 pm and 4 am compared to 10 am−10 pm (37). Lipids increase in the morning, reach a peak from midday to evening, and reach lower values at night (38–40). Ghrelinisalso present in human milk as well as insulin, adiponectin, obestatin, and other metabolic hormones (15, 41). No diurnal differences have been found among these latter hormones or in total proteins, carbohydrates, and lactose, or there are controversies about their diurnal differences (42, 43). However, of 17 amino acids explored, only a clear rhythm of tryptophan was observed in colostrum, transitional, and mature human milk with an acrophase at approximately 0400 h, and then its levels decrease and reach a nadir during the afternoon (7). In mature milk, clear rhythms were detected also in methionine, aspartic acid, histidine, phenylalanine, and tyrosine, with an acrophase at different times of the day (7). In mature milk, there are also rhythms of nucleotides, adenosine 5'monophosphate (5'AMP), guanosine 5'monophosphate (5'GMP), uridine 5'monophosphate (5'UMP),cytidine5'monophosphate (5'CMP) and inosine 5'mono‘hosphate (5'IMP), the first three with higher levels during the night and the latter two during the day (44). In mature milk, 2-AG showed significantly higher concentration during the day than during the night, which mirrored plasma levels in the mother [(33); Figure 2A].

## Diurnal Changes in Immunological Factors

Earlier studies reported no differences in concentration of IgA, IgM, and IgG between day and night (46, 47). Further, in a study of 36 mothers, colostrum, transitional and mature human milk, sIgA was higher during the day at 12:00 h than during the night at 24:00 h. IgG and IgM were also higher during the day than during the night in transitional and mature milk [(48); but see (19)]. C3 and C4 complement proteins, which opsonize pathogens and participate in the innate and adaptive response, increased significantly in colostrum during the diurnal period in humans (48). There are also reports about significant differences between day and night concentrations of cytokines IFNγ, IL4, IL5, and IL10 (49, 50). No diurnal differences have been found in Lactoferrin concentrations (46).

## Physiological Importance of Diurnal Changes in Milk Components

During late pregnancy the fetus expresses rhythms of total activity, heart rate, and general and breathing movements (51–53). This is not only a response to maternal rhythms, as the fetal brain is necessary for the integration of the mother's cues (54). Disruption of circadian rhythms during pregnancy leads to an array of negative pregnancy outcomes, such as increased frequency of miscarriage, preterm infant delivery, and low birth weight (55). Upon birth the newborn is exposed to a variety of manipulations and environmental changes and time of the establishment of circadian rhythms of CORT, MEL and day/night rest and sleep rhythm vary widely at 3–6 months of age (56). In a study in which the infant was breastfed on demand during the day and night and exposed to light only during day time, the circadian rhythms of temperature, sleep/wake, and MEL were detected during the first week and at 30 and 45 days of life, respectively (57), significantly earlier than most reports. Accumulating evidence reveals the benefits of changes in milk components and environmental conditions for the infant.

The higher concentration of tryptophan and nucleotides 5'AMP, 5'GMP, and 5'UMP may play an important role as sleep promoters. It has been established that adenosine decreases cellular activity and is a somnogen (58). In agreement, there is an increase of 5'GMP in adult human plasma during sleep in the night in comparison to the period of wakefulness (59). In human milk, the concentration of 5'AMP, 5'GMP, and 5'UMP is higher during the night than during the day (44). The effect of the differential concentration of tryptophan and the nucleotides was tested in children. Sleep-wake cycle in newborns is not established and they nurse during the day and the night (60). They ingest tryptophan and nucleotides during the night, when the concentrations are highest. Tryptophan is a precursor of the hormone melatonin, and its ingestion corresponds with the variable levels of 6-sulfatoxymelatonin in urine, a metabolite of melatonin (61). To test the effect of this amino acid on sleep, tryptophan was added to formula milk, along with 5'AMP and 5'UMP to children 4–20 weeks of age. Children that received formula with this amino acid and nucleotides from 18:00 to 06:00 h showed a reduction in their latency to sleep as well as an increase in their hours of sleep (62). In another study of the same group, 8–16 month old children were fed a cereal enriched with tryptophan, 5'AMP, and 5'UMP during the night. Actigraphic recording revealed an improvement in sleep parameters (63). These experiments support the assumption that tryptophan, 5'AMP, 5'UMP, and MEL in human milk during the night improve infant sleep and help to consolidate their sleep/wake cycle. Vitamin B12 also may improve sleep in children. In humans, it has been associated with the modulation of sleep and circadian rhythms (64–66), and its deficiency during pregnancy has been associated with a subsequent increase in the infants' crying (67). Together the evidence indicates that there are chronobiotic substances in milk that contribute to the establishment of the sleep-wake cycle of the infant. Indeed, a recent study demonstrates that breastfed infants achieved a circadian rest-activity rhythm at 6th week age in contrast to 12 weeks in mixed, formula and breastmilk-fed babies (68). Exclusively breastfed infants had better sleep parameters in comparison to formula-fed infants (69). Infants at 2 months of age who were breastfed, in contrast to formula-fed infants, had a significantly lower frequency of colic attacks and severity of irritability attacks, which was associated with the night-time consumption of MEL through milk (70).

MEL has a strong interaction with the immune system. Human lymphocytes contain membrane MEL receptors which suggest that they can detect physiological changes in this hormone (71). *In vitro* studies have demonstrated an effect of MEL on the phagocytic activity of mononuclear and polymorphonuclear lymphocytes from colostrum. When exposed to *Escherichia coli*, in the presence of MEL, these cells increase their phagocytic, and bactericidal activity by stimulating cellular oxidative metabolism (6). In another example, TNF-α, a regulator of inflammatory processes, which is present in human milk, inhibits the *in vitro* synthesis of MEL in the ratpineal gland (72). This information is relevant, as cesarean section delivery in humans increased the production of TNF-α in colostrum, in parallel with a suppression of the nocturnal MEL increase (50). This can lead to an inflammatory process and to a disruption of the beneficial actions of MEL in the newborn. There are reports that the addition of nucleotides to formula milk significantly increased weight gain (73) and the rate of the occipitofrontal head circumference gain in infants 2 months of age (74). Infants fed with milk supplements enriched with DHA had a lower incidence of bronchiolitis and bronchitis during the first year of age (75).

The high level of 2-AG cannabinoids in human milk may modulate the infant's food intake, and this also can be influenced by the weight of the mother. Milk of overweight and obese mothers has a larger concentration of 2-AG and this may have an impact on the body mass index of the infant because of the effect of endocannabinoids on food intake and the hedonic properties of food (33, 76, 77). Overweight mothers give birth to overweight babies (78). Cannabinoids are involved in development, as the administration of an antagonist of the CB1 receptor within the first day after birth in mice; inhibited milk ingestion due to an impairment of the pup's suckling response (79). This evidence and that cannabinoids are one of the few compounds in human milk with diurnal variation; suggest that they play an important role for the infant. It is noteworthy that breastfeeding also has important benefits for the mother, as it reduces the risk of ovarian cancer, mammary cancer, and postpartum depression (80).

## Effects of Environmental Light/Dark Cycle on Neonates

The importance of circadian rhythms for the wellbeing of infants has been reported in preterm infants in a neonatal intensive care unit. After parturition, premature infants were separated from their mothers and usually did not ingest their mother's milk. The infant's pineal in humans does not secrete MEL, and those infants were maintained in constant light conditions (81). This strategy severely affected their survival. Infants in this facility maintained in constant light had a lower weight gain, spent more days on the ventilator and on phototherapy, displayed lower motor coordination, and showed a delayed response to be fed orally, in contrast to babies maintained in a cycled light-dark condition (81). In another study, a personalized helmet over the head of the infant that covered their eyes but permitted airflow was used to maintain 12:12 h, light/dark condition, and infants were fed with mother's milk. Infants under this procedure showed faster weight gain, improved oxygen saturation, more rapidly developed a melatonin rhythm, and attained a shorter discharge stay in the hospital [(45); Figure 2B]. These studies are examples of the importance of circadian rhythms after birth in infant. Fortunately, the compelling evidence of the benefits of exposing premature infants to a light/dark cycle is leading neonatal care societies to recommend this practice for clinical applications (82).

## Conclusion

The complexity of milk stands in sharp contrast to the concept of simple food for the infant. The analysis of milk components and their diurnal changes has led to the enrichment of milk substitutes. Unfortunately, there is only limited research on the role of breastfeeding and control of light-dark conditions in the establishment of infant circadian rhythms and wellbeing. Based on the evidence reviewed herein, we consider this issue to deserve attention, as regarding infant nutrition, there is increasing use of milk supplements, which are devoid of myriad nutritive and non-nutritive components, and lack the diurnal rhythms inherent in breastmilk. Long-term studies demonstrate that breastfeeding, in contrast to infant formula, has multiple benefits: decreased risk of developing obesity and type 2 diabetes, gastrointestinal, ear, and respiratory tract infections, and improved cognitive neurodevelopment and mental and behavioral health (83–85). Many of these benefits are epigenetic, *via* the components of breast milk, including the infant's microbiome (86). In a recent survey of 130 countries, it was concluded that suboptimal breastfeeding causes ~600,000 child deaths/year primarily from pneumonia and diarrhea (87). Moreover, breastfeeding since birth is effective in preventing death in premature babies, in contrast to conventional care practices (88). A milk substitute cannot replace the complex composition and diurnal dynamic changes of breastmilk, so major approaches must be developed to promote the benefits of breastfeeding over commercial formula to optimize infant nutrition and subsequent health. Thus, the control of the light/dark conditions (89) in addition to the circadian variations in milk components through exclusive breastfeeding every 2–5 h (57), is strongly recommended as an important strategy to improve health and proper infant development.

## Author Contributions

MC-F, AR-L, AC-M, CM-V, RV-C, and MC contributed to the conception, writing, and figures design. All authors approved the submitted version of this article.

## Funding

This study was funded by the COVEICYDET (151746) to MC.

## Conflict of Interest

The authors declare that the research was conducted in the absence of any commercial or financial relationships that could be construed as a potential conflict of interest.

## Publisher's Note

All claims expressed in this article are solely those of the authors and do not necessarily represent those of their affiliated organizations, or those of the publisher, the editors and the reviewers. Any product that may be evaluated in this article, or claim that may be made by its manufacturer, is not guaranteed or endorsed by the publisher.
